# Impact of multidisciplinary Endocarditis Team on management of infective endocarditis

**DOI:** 10.1016/j.bjid.2024.103870

**Published:** 2024-09-20

**Authors:** Nicolas Fourré, Virgile Zimmermann, Benoit Guery, Nicoleta Ianculescu, Piergiorgio Tozzi, Matthias Kirsch, Pierre Monney, Matthaios Papadimitriou-Olivgeris

**Affiliations:** a Lausanne University Hospital and University of Lausanne, Infectious Diseases Service, Lausanne, Switzerland; b Lausanne University Hospital and University of Lausanne, Department of Cardiology, Lausanne, Switzerland; c Lausanne University Hospital and University of Lausanne, Department of Cardiac Surgery, Lausanne, Switzerland; d Cantonal Hospital of Sion and Institut Central des Hôpitaux (ICH), Infectious Diseases Service, Sion, Switzerland

**Keywords:** Infective endocarditis, Valve surgery, Endocarditis-Team, Multidisciplinary management, ^18^F-FDG PET/CT, Embolic events

## Abstract

Infective Endocarditis (IE) is a complex, life-threatening disease. The aim of the present study was to evaluate the impact of the Endocarditis-Team on management of IE. This observational study conducted at a university hospital (2015‒22), included adult patients with IE. The study period was divided in two periods: before (pre-Endocarditis-Team; pre-ET) and after the establishment of the Endocarditis-Team (post-Endocarditis-Team; post-ET) on January 2018. Among 505 IE episodes (187 in pre-Endocarditis-Team, 318 in post-ET period), ^18^F-Fluorodeoxyglucose Positron Emission Tomography/Computed Tomography was more commonly used in post-ET period (14 % vs. 28 %; *p* < 0.001). Overall, thirty-day and one-year mortality were 14 % and 27 %, respectively; no difference was observed between the two periods. In post-ET period, the administration of 4-weeks, rather than 6-weeks, of intravenous antimicrobial treatment was higher than in the post-ET period (15 % vs. 45 %; *p* < 0.001). Indication for surgery was present in 115 (61 %) patients in pre-ET and in 153 (48 %) in the post-ET period. In post-ET period, among patients with indication, valve surgery was more frequently performed (66 % vs. 78 %; *p* = 0.038). Such difference was due to a higher acceptance of operative indication by the cardiac surgeon (69 % vs. 94 %; *p* = 0.013). The observed increase in number of patients benefiting from cardiac surgery in the post-ET period led to a decrease of subsequent embolic events, since among patients with operative indication (*n* = 268), new embolic events after the establishment of the indication were more common in the pre-ET period compared to post-ET (23 % vs. 12 %; *p* = 0.033). After the implementation of the multidisciplinary Endocarditis-Team we observed several improvements in the general management of IE patients.

## Introduction

Infective Endocarditis (IE) can present with a wide range of symptoms and signs diagnosis can be challenging, as patients often present with nonspecific symptoms.^1^^,^^2^ Diagnosis is based on a combination of clinical symptoms/signs, microbiologic tests, including mainly blood cultures, and imaging studies. Several attempts to establish clinical criteria to diagnose IE were previously undertaken. Since their introduction in 1994, the Duke criteria and their subsequent revisions were the mainstay of diagnosis.^1^^,^^3^^,^^4^ However, those criteria were established for research purposes and their performance in the clinical setting remained suboptimal, especially among patients with prosthetic valve IE, or among patients with negative blood cultures due to prior antimicrobial treatment.

IE is a rare and complex disease associated with significant morbidity and mortality.^1^^,^^2^ Prompt identification of IE and its complications is essential for improving prognosis, since rapid establishment of appropriate antimicrobial treatment and prompt interventions such as valve surgery or Cardiac Implantable Electronic Device (CIED) removal when indicated, were associated with better outcome.^1^^,^^5^, ^6^, ^7^ Valve surgery is required in 40 %‒50 % of IE patients; the principal indications being acute heart failure due to acute valvular failure, uncontrolled infection and prevention of embolic events.^1^ The timing of surgery is critical and should be individualized based on the patient's status and the severity of the infection, with emergent surgery being recommended in patients with refractory pulmonary oedema or cardiogenic shock.^1^

Based on the complexity of diagnosis and management of IE patients, the 2015 European Society of Cardiology (ESC) guidelines recommended a multidisciplinary approach for the optimal management of such patients.^1^ The same recommendation remained also in the revised guidelines of 2023.^8^ The implementation of Endocarditis-Team was shown to increase the rate of surgical intervention and reduce mortality,^1^^,^^9^^,^^10^ but these results were not universally found.^11^, ^12^, ^13^, ^14^

The aim of our study was to assess the impact of an Endocarditis Heart-Team approach on the diagnosis and management of IE by performing a before-and-after analysis.

## Materials and methods

### Study design

The study was conducted at a university hospital, a 1100-bed primary and tertiary care hospital from January 2015 to June 2022 (2015–17: retrospective cohort, 36-months; 2018 onwards: prospective cohort, 54-months). The study was approved by the ethics committee of the Canton of Vaud (CER-VD 2017 02137).

### Patients

Inclusion criteria were adult patients (≥ 18-years-old) and diagnosis of IE. Additional inclusion criterion for the prospective cohort was the written consent and for the retrospective cohort the absence of refusal of the use of their data. Patients that were transferred from another hospital after 72 h from hospitalization were excluded. A subsequent episode was excluded if it occurred within one-year from the initial one. All patients are followed for at least 1-year from IE diagnosis.

Data regarding demographics (age, sex), comorbidities, cardiac predisposing factors,^13^ CIEDs, microbiologic etiology, systemic symptoms, fever, acute heart failure, sepsis or septic shock, heart murmur, immunological phenomena,^13^ cardiac and non-cardiac imaging studies, site of cardiac involvement and type of lesion, cardiac surgery (timing, indication), embolic events (type, timing) and antimicrobial treatment were retrieved from patients’ electronic health records.

### Management of IE

An Endocarditis-Team was established on January 2018, including infectious diseases specialists, cardiologists, and cardiac surgeons, which reviewed all patients with suspected IE suspicion during weekly meetings. Additionally, microbiologists, radiologists and specialists in nuclear medicine participated when indicated.

According to internal guidelines (before and after Endocarditis-Team establishment), an infectious diseases consultation with a thorough physical examination was performed on a mandatory basis for all patients with suspected IE. Thoraco-abdominal and cerebral imaging studies were performed in all symptomatic patients.^15^^,^^16^ Their realization in asymptomatic patients was left at the discretion of the treating physician and infectious diseases consultant.

### Definitions

The study period was divided in two periods; the one before (pre-ET; from 2015 to 2017) and the other after the implementation of Endocarditis-Team (post-ET; from 2018 to 2022). In both periods, the diagnosis of IE was made on day 60 according to the 2015 ESC modified Duke criteria.^13^ Indications for valve surgery were also based on the aforementioned guidelines.^13^ The date of establishment was defined as the day on which an episode fulfilled any of the criteria outlined in the guidelines.^13^

### Endpoints

The primary endpoint was 30-day (early) mortality. Secondary endpoints were one-year (late) mortality, realization of cardiac and non-cardiac imaging studies, realization of valve surgery when indicated, new embolic events after the establishment of operative indication and adherence to guidelines for the choice and duration of antimicrobial treatment.

### Analysis

SPSS version 26.0 (SPSS, Chicago, IL, USA) software was used for data analysis. Categorical variables were analyzed using the Chi-Square or Fisher's exact test and continuous variables with Mann-Whitney *U* test. Based on the 2015 ESC guidelines, a duration of 4 to 6 weeks of IV antimicrobial treatment is indicated for native valve IE.^13^ We evaluated the duration of IV antimicrobial treatment in patients who did not require treatment for >4-weeks. For this analysis, we excluded patients with prosthetic valve IE, CIED-IE only, enterococcal IE treated with amoxicillin-ceftriaxone combination. non-cardiac infectious complications requiring IV antimicrobial treatment exceeding 4-weeks (such as cerebral or epidural abscesses), and those who died before completing 4-weeks of treatment. All statistic tests were 2-tailed and *p* < 0.05 was considered statistically significant.

## Results

**Fig. 1:**
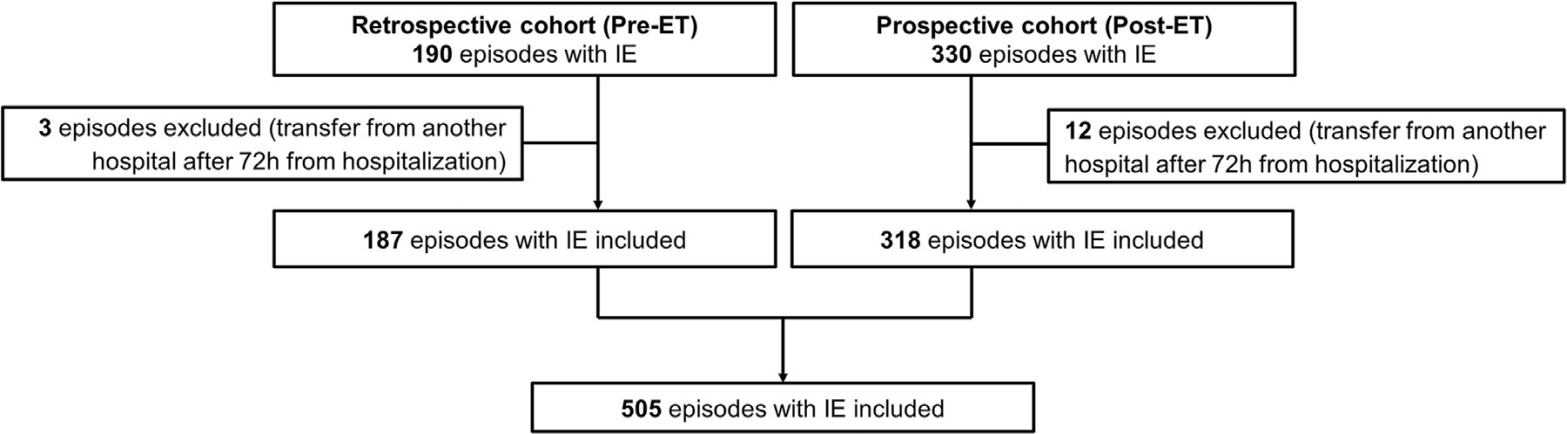
Flowchart of included patients. ET, Endocarditis-Team.

Among 520 IE episodes, 505 were included (15 episode were excluded since patients were transferred from another hospital after 72 h from hospitalization); 187 in the pre-ET (5.2 IE episodes per month) and the remaining 318 in post-ET period (5.9 per month) (Fig. 1).

The comparison of IE patients in pre-ET and post-ET patients is shown in Table 1. Patients in post-ET were older and had higher Charlson Comorbidity Index compared to pre-ET. No difference on microbiological aetiology, manifestations, site of intracardiac infection or type of intracardiac lesions was observed between the two periods.

**Table 1:** Characteristics of IE patients in pre-ET and post-ET periods.

	Pre-ET (*n* = 187)		Post-ET (*n* = 318)		p
Demographics					
Male sex	145	78 %	238	75 %	0.520
Age (years)	65	50‒75	68	53‒77	0.068
Age > 60-years	110	59 %	217	68 %	0.034
Co-morbidities					
Congestive heart failure	11	6 %	45	14 %	0.005
Chronic obstructive pulmonary disease	26	14 %	36	11 %	0.402
Cirrhosis	10	5 %	22	7 %	0.573
Diabetes mellitus	39	21 %	85	27 %	0.164
Chronic kidney disease (moderate or severe)	29	16 %	61	19 %	0.336
Malignancy (solid organ or haematologic)	9	5 %	40	13 %	0.005
Obesity	39	21 %	77	24 %	0.443
Immunosuppression	19	10 %	28	9 %	0.636
Charlson Comorbidity Index	3	1‒6	5	2‒7	0.007
Charlson Comorbidity Index > 4	69	37 %	160	50 %	0.004
Transfer from other hospital (within 4-days from diagnosis)	52	28 %	108	34 %	0.166
Setting of infection onset					
Community or non-nosocomial healthcare-associated	164	88 %	278	87 %	
Nosocomial	23	12 %	40	13 %	1.000
Cardiac predisposing factors	95	51 %	155	49 %	0.712
Cardiac implantable electronic devices	26	14 %	70	22 %	0.026
Microbiological data					
*S. aureus*	77	41 %	126	40 %	0.778
Coagulase negative staphylococci	12	6 %	25	8 %	0.600
Streptococci	45	24 %	84	26 %	0.598
Enterococci	27	14 %	39	12 %	0.496
Other Gram-positive	5	3 %	12	4 %	0.615
HACEK	10	5 %	8	3 %	0.134
Other Gram-negative	3	2 %	9	3 %	0.549
Intracellular pathogens	1	1 %	4	1 %	0.656
Fungi	3	2 %	3	1 %	0.675
Polymicrobial infection	6	3 %	7	2 %	0.564
No identification	10	5 %	15	5 %	0.832
Manifestations					
Fever	155	83 %	252	79 %	0.352
Heart murmur	119	64 %	183	58 %	0.189
New heart murmur	77	41 %	135	42 %	0.852
Immunologic phenomena	14	7 %	28	9 %	0.462
Sepsis	78	42 %	142	45 %	0.577
Septic shock	35	19 %	49	15 %	0.386
Embolic event	115	61 %	168	53 %	0.064
Embolic event after introduction of antibiotic treatment	65	35 %	95	30 %	0.276
Embolic event after establishment of operative indication (*n* = 268)	26	23 %	19	12 %	0.033
Cardiac imaging					
TTE	176	94 %	290	91 %	0.301
TOE	148	79 %	256	81 %	0.730
^18^F-FDG PET/CT	26	14 %	88	28 %	<0.001
Cardiac-CT	10	5 %	21	7 %	0.702
Non-cardiac imaging studies					
Thoracoabdominal imaging	147	79 %	257	81 %	0.566
Thoracoabdominal imaging in asymptomatic patients	65	35 %	156	49 %	0.002
Cerebral imaging	114	61 %	206	65 %	0.391
Cerebral imaging in asymptomatic patients	48	26 %	102	32 %	0.132
Site of infection					
Aortic valve	93	50 %	160	50 %	0.927
Mitral valve	75	40 %	132	42 %	0.779
Other left-side site of infection	4	2 %	0	0 %	0.018
Tricuspid valve	19	10 %	28	9 %	0.636
Pulmonary valve	5	3 %	6	2 %	0.546
Multivalvular	16	9 %	36	11 %	0.365
CIED-IE	14	7 %	39	12 %	0.100
Type of valve					
Native	129	69 %	215	68 %	0.768
Prosthetic	48	26 %	79	25 %	0.833
Type of intracardiac lesions					
Vegetation	133	71 %	201	63 %	0.080
Vegetation ≥ 10 mm	72	39 %	114	36 %	0.567
Abscess	31	17 %	66	21 %	0.293
Other intracardiac lesions^a^	8	4 %	10	3 %	0.620
Intervention					
Valvular surgery in presence of operative indication (*n* = 268)					
Operative indication with surgery	76	66 %	119	78 %	0.038
Operative indication without surgery	39	34 %	34	22 %	
Operative indication not retained by cardiac surgeon (*n* = 73)	12	31 %	2	6 %	0.013
Timing from operative indication to surgery (days; *n* = 195)	5	2‒8	3	1‒7	0.239
CIED extraction (*n* = 54 patients with CIED-IE or operated valvular IE)	16	94 %	38	76 %	0.158
Duration of IV antibiotic treatment (*n* = 274)^b^					
4-weeks	16	15 %	73	45 %	
More than 4-weeks	94	85 %	91	55 %	<0.001
30-day mortality	28	15 %	42	13 %	0.595
1-year mortality	54	29 %	83	26 %	0.534

Data are depicted as number/percentage or median/Q_1_‒Q_3_.

^18^F-FDG PET/CT ^18^F-Fluorodeoxyglucose Positron Emission Tomography/Computed Tomography; CIED, Cardiac Implantable Electronic devices; ET, Endocarditis Team; HACEK, *Haemophilus* spp, *Aggregatibacter* spp, *Cardiobacterium hominis, Eikenella corrodens, Kingella kingae*; IE, Infective Endocarditis; TTE, Transthoracic Echocardiography; TOE, Transesophageal Echocardiography.

a Perforation, dehiscence of prosthetic valve, fistula, aneurysm, pseudoaneurysm.

b After excluding patients with prosthetic valve IE, CIED-IE only, enterococcal IE treated with amoxicillin-ceftriaxone combination, other infectious complication warranting IV antimicrobial treatment > 4-weeks, and those deceased before 4-weeks.

**Fig. 2:**
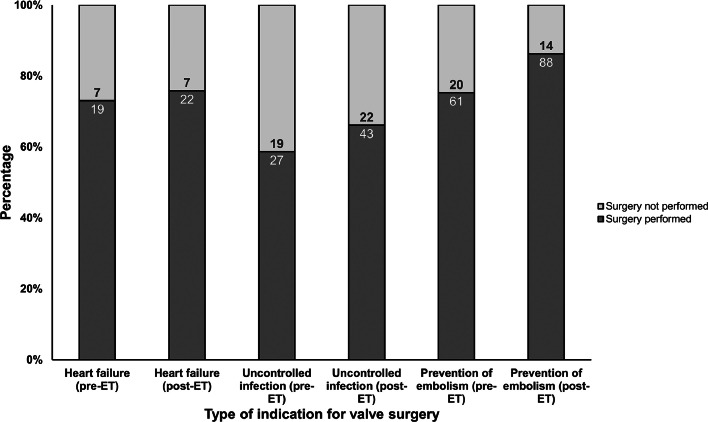
Performance of valve surgery among patients that valve surgery was warranted. ET, Endocarditis-Team.

Thirty-day and one-year mortality were 14 % and 27 %, respectively. No difference on early and late mortality was observed between pre-ET and post-ET periods. In post-ET period, the administration of 4-weeks of IV antimicrobial treatment was higher than in the pre-ET (15 % vs. 45 %; *p* < 0.001). Indication for surgery was present in 115 (61 %) patients in pre-ET and in 153 (48 %) post-ET. In post-ET, valve surgery was more frequently performed (66 % vs. 78 %; *p* = 0.038) among patients with indication (Fig. 2). Such difference was due to a higher acceptance of operative indication by the cardiac surgeon in the post-ET period (69 % vs. 94 %; *p* = 0.013). Among patients with operative indication (*n* = 268), new embolic events after the establishment of the indication were more common in the pre-ET period compared to post-ET period (23 % vs. 12 %; *p* = 0.033).

**Fig. 3:**
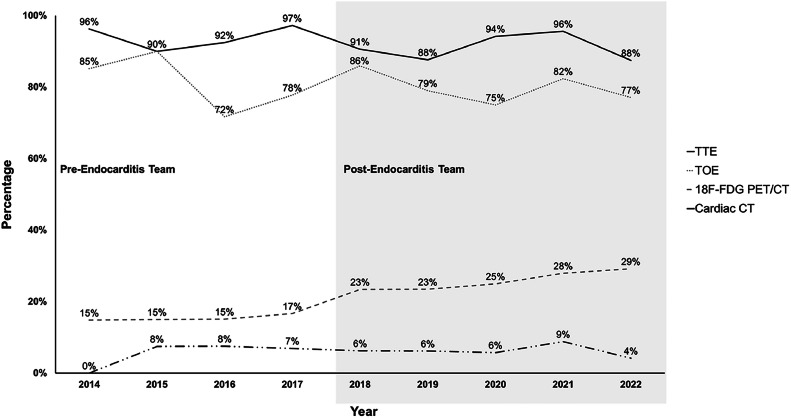
Percentage of patients benefiting from cardiac imaging studies. ^18^F-FDG PET/CT, ^18^F-Fluorodeoxyglucose Positron Emission Tomography/Computed Tomography; TTE, Transthoracic Echocardiography; TOE, Transesophageal Echocardiography.

No difference was observed between the two periods on rate of performance of transthoracic or transesophageal echocardiograms, cardiac CT and thoracoabdominal or cerebral imaging studies (Fig. 3). In post-ET period, ^18^F-Fluorodeoxyglucose Positron Emission Tomography/Computed Tomography (^18^F-FDG-PET/CT) was more commonly used (14 % vs. 28 %; *p* < 0.001).

## Discussion

The present study demonstrated improvements in the management of IE patients (diagnosis, antibiotic treatment, surgery) after the introduction of an Endocarditis-Team.

Despite the improvement in diagnosis, medical and surgical management in the post-ET period, we did not find an improvement on survival. Our study showed comparable mortality rates to previous ones.^11^, ^12^, ^13^, ^14^^,^^17^ Only three of the aforementioned studies found a decrease in mortality in post-ET period;^9^,^17^,^18^ in one after applying a propensity score the impact on mortality dissipated.^17^ In a meta-analysis of studies on management of IE, the implementation of multidisciplinary teams was associated with decreased short-term mortality.^19^ The absence of impact of the Endocarditis-Team on mortality in the present study, can be explained by the fact that in the pre-ET period, all IE patients were followed by an infectious diseases consultant who acted as the intermediary for other consultants such the cardiologist and cardiac surgeon, thus a more informal type of “Endocarditis-Team” existed before the creation of the official Endocarditis-Team. In most of previous studies, no information on the management of IE patients in the pre-ET period was mentioned.^9^^,^^11^^,^^12^^,^^14^^,^^17^^,^^18^^,^^20^, ^21^, ^22^ In our institution, infectious diseases consultation among patients with *Staphylococcus aureus* bacteraemia, of which 14 % had IE, was associated with better outcome, highlighting the importance of such intervention.^23^

The main finding of the present study was an increase in the number of valve surgery performed among patients with an operative indication.^24^ The observed increase in valve surgery in the post-ET period was due to higher acceptance of operative indication by the team of cardiac surgery during the weekly meetings. The indications that were dismissed by cardiac surgeons in the pre-ET related to prevention of embolism. Such indications have minimal effect on mortality, but can impact morbidity by decreasing further embolic events.^25^ Previous studies showed no significant increase on surgical management,^9^^,^^12^^,^^13^^,^^17^^,^^18^^,^^20^, ^21^, ^22^ but some exhibited that valve surgery in the post-ET period was performed earlier than in the pre-ET.^12^^,^^17^ In the present study, even though valve surgery was performed earlier in the post-ET period (3-days from operative indication establishment) compared to pre-ET period (5-days), this did not achieve statistical significance (*p* = 0.239). Another explanation for the lack of impact of the Endocarditis Team on mortality in the present study might be that even in the pre-ET period, patients were operated on earlier than reported in prior studies (median of 5-days vs. 6‒14).^6^^,^^13^^,^^24^

The increase in valve surgery in the post-ET period could explain the decrease in further embolic events observed in the present study.^13^ No study to date evaluated the role of Endocarditis-Team in outcomes other than mortality or length of stay. By investigating a wider range of outcomes beyond mortality, we could gain a more comprehensive understanding of the effectiveness of the multidisciplinary approach in managing infective endocarditis.

Another finding was the observed shortening on the antibiotic treatment duration. The 2015 ESC guidelines propose that among patients with native valve IE due to staphylococci and enterococci a duration of 4 to 6 weeks, and for streptococci 4-weeks.^1^ We noted that in patients with native valve IE not necessitating an extension of IV treatment beyond 4-weeks, there was an increase in the proportion of individuals receiving a 4-week course of IV antibiotic therapy, from 15 % in the pre-ET to 45 % in the post-ET (*p* < 0.001). The duration of antibiotic treatment was seldomly reported in previous studies,^12^^,^^20^ with conflicting results; one study showed no difference on antibiotic treatment duration,^20^ while another showed a significant decrease of antibiotic treatment duration in the post-ET,^12^ and in a third all patients in pre- and post-ET periods received appropriate antimicrobial duration.^24^

The establishment of the Endocarditis-Team did not impact the rates of transthoracic and transoesophageal echocardiograms, which were high in both periods. In two studies, an increase in transoesophageal echocardiograms was found in post-ET.^9^^,^^11^ Following evidence regarding an improvement in the diagnosis of prosthetic valve IE by ^18^F-FDG PET/CT and the recommendation of the 2015 ESC guidelines,^1^^,^^26^^,^^27^ an increase in the realization of aforementioned imaging study was observed in the post-ET period. While between 2014 and 2017, the utilization of ^18^F-FDG PET/CT ranged from 15 % to 17 %, this rate increased to 23 % in 2018. Such an increase in ^18^F-FDG PET/CT in the post-ET was also observed in a previous study.^9^ The 2015 ESC guidelines also recommend considering non-cardiac imaging studies (thoracoabdominal or cerebral) for the detection of embolic events in patients with a high clinical suspicion but for whom IE diagnosis is not yet proven, even though they might not offer a diagnostic advantage.^1^^,^^15^ In the present study, Endocarditis-Team did not influence the rate of such imaging studies.

Another role of the Endocarditis Team extended beyond the management of IE patients to include research activities. The team adjudicated whether patients with suspected IE, had or not IE based on microbiological, clinical, imaging, surgical, and pathological findings presented at weekly meetings. This process served as a reference standard for evaluating different versions of the Duke criteria and various prediction scores used to diagnose IE in patients with bacteremia caused by typical microorganisms.^28^, ^29^, ^30^

Our study has several limitations. First, this is a single-center, observational study, with a moderate number of patients, even though in the present study the study size was significantly higher than most previous studies.^9^^,^^11^^,^^12^^,^^14^^,^^17^^,^^18^^,^^20^, ^21^, ^22^ The difference of type of inclusion could offer a bias, since after 2018 patients were included in a prospective manner. In the prospective cohort, 88 % of eligible patients provided informed consent and were consequently included in the study. Similarly, in the retrospective cohort, 91 % of eligible patients were included, with only 9 % having not sign the general informed consent. This high inclusion rate suggests a robust representation of patients in both cohorts, minimizing potential biases related to patient selection. Second, since all patients in the pre-ET were followed by an infectious disease's specialist, the real impact of an Endocarditis-Team approach may be underestimated. Therefore, the present results must be generalized with caution. Last, patients in the pre-ET period were included retrospectively; in order to minimize the bias, patients with IE were identified by three different approaches: 1) ICD-10 coding in the discharge letter, 2) Cardiac surgery and CIED-removal, and 3) Bacteraemia by typical IE pathogens. Another limitation was that some of the differences observed between the two time periods could be explained due to advances on IE diagnosis of IE; however, concerning ^18^F-FDG PET/CT, there was an abrupt increase in 2018, probably attributable to the Endocarditis-Team presence.

## Conclusions

The implementation of a multidisciplinary Endocarditis-Team offered several improvements in the overall management of IE patients, which included increased utilization of advanced imaging studies, such as ^18^F-FDG PET/CT, a reduction in the duration of IV antimicrobial treatment and expansion of the number of patients benefiting from cardiac surgery. Although these changes did not have a discernible impact on early or late mortality, they did lead to a significant decrease in subsequent embolic events attributed to a higher number of patients undergoing valve surgery. To comprehensively evaluate the impact of a multidisciplinary Endocarditis-Team, it is imperative to conduct further prospective, multicenter studies that explore a wide range of outcomes beyond mortality.

## Data availability

The data that support the findings of this study are available from the corresponding author upon reasonable request.

## Authors’ contributions

MPO and PM conceived the idea. NF, VZ, BG, NI, PT, MK and MPO collected the patients' data. PM supervised the project. MPO performed the analysis and interpreted the results. NF and VZ wrote the manuscript. All authors contributed to manuscript revision and read and approved the submitted version.

## Conflicts of interest

The authors declare no conflicts of interest.
